# Rethinking Chronic Kidney Disease in the Aging Population

**DOI:** 10.3390/life12111724

**Published:** 2022-10-28

**Authors:** Gaetano Alfano, Rossella Perrone, Francesco Fontana, Giulia Ligabue, Silvia Giovanella, Annachiara Ferrari, Mariacristina Gregorini, Gianni Cappelli, Riccardo Magistroni, Gabriele Donati

**Affiliations:** 1 Nephrology Dialysis and Transplant Unit, University Hospital of Modena, 41124 Modena, Italy; 2 General Medicine and Primary Care, University of Modena and Reggio Emilia, 41124 Modena, Italy; 3 Surgical, Medical and Dental Department of Morphological Sciences, Section of Nephrology, University of Modena and Reggio Emilia, 41124 Modena, Italy; 4 Clinical and Experimental Medicine Ph.D. Program, University of Modena and Reggio Emilia, 41124 Modena, Italy; 5 Nephrology and Dialysis, AUSL-IRCCS Reggio Emilia, 42122 Reggio Emilia, Italy

**Keywords:** chronic kidney disease, aging, dialysis, palliative care, glomerular filtration rate, creatinine, proteinuria, renal replacement therapy, RAAS, SGLT-2, finerenone, senescence

## Abstract

The process of aging population will inevitably increase age-related comorbidities including chronic kidney disease (CKD). In light of this demographic transition, the lack of an age-adjusted CKD classification may enormously increase the number of new diagnoses of CKD in old subjects with an indolent decline in kidney function. Overdiagnosis of CKD will inevitably lead to important clinical consequences and pronounced negative effects on the health-related quality of life of these patients. Based on these data, an appropriate workup for the diagnosis of CKD is critical in reducing the burden of CKD worldwide. Optimal management of CKD should be based on prevention and reduction of risk factors associated with kidney injury. Once the diagnosis of CKD has been made, an appropriate staging of kidney disease and timely prescriptions of promising nephroprotective drugs (e.g., RAAS, SGLT-2 inhibitors, finerenone) appear crucial to slow down the progression toward end-stage kidney disease (ESKD). The management of elderly, comorbid and frail patients also opens new questions on the appropriate renal replacement therapy for this subset of the population. The non-dialytic management of CKD in old subjects with short life expectancy features as a valid option in patient-centered care programs. Considering the multiple implications of CKD for global public health, this review examines the prevalence, diagnosis and principles of treatment of kidney disease in the aging population.

## 1. Introduction

Since 2013, the World Health Organization (WHO) has endorsed a project called “The Global Action Plan” aimed to promote health and psychophysical well-being of subjects worldwide. The goal of this program is to reduce mortality due to non-communicable diseases by 25% in 2025 through a wide action of prevention and control of risk factors [1].

Chronic kidney disease (CKD) is recognized as a major public health problem affecting >10% of the global population. CKD is highly associated with morbidity and mortality and is linked to numerous negative effects that can potentially aggravate the outcome of the five most serious diseases (i.e., cardiovascular disease, diabetes, high blood pressure, HIV and malaria). Although CKD is not listed among the most clinically relevant chronic diseases, its management deserves careful consideration, because it has the potential to affect the prognosis of subjects and impact heavily on the resources of national healthcare systems [2]. 

CKD is a progressive chronic disease, leading to end-stage kidney disease (ESKD) in 1–2% of cases [3,4]. The Global Burden of Disease Study, an epidemiological study conducted in 195 countries, showed that kidney disease was responsible in 2015 for disability and reduced life expectancy by a cumulative 18 and 19 million years, respectively. The total number of reported death due to kidney disease accounted for 1.2 million yearly. This estimate was expected to rise to about 3–5 million/year people if mortality due to acute renal failure was included. However, the extent of the problem is likely underestimated, as access to laboratories for the screening of CKD is precluded in some geographical areas [5].

Kidney disease has the potential to involve all ages but is principally prevalent in older individuals. Aging is closely associated with kidney injury as advancing age is the major risk factor for kidney disease and age-related comorbidities. Aging is also implicated in the deleterious changes of kidney parenchyma secondary to cellular senescence as well as to cumulative effects of nephrotoxic agents prescribed during the patient’s life [6,7]. According to the latest projections, every country in the world is experiencing a rise in the number of older persons in their population. This subset of the population, at high risk of developing CKD, is projected to double in 2050 when one in six people in the world will be aged 65 years or over [8]. The current demographic transition will raise the prevalence of people with CKD. Consequently, the demand for care will pose a particular challenge to the concerning use of vast human and financial resources.

Based on this background, this review aims to summarize recent literature on the epidemiology of CKD and furnish the reader with the key elements for managing kidney disease. We propose the concept of the age-adapted definition of CKD in order to undertake an appropriate and calibrated risk stratification of kidney disease in the setting of this extraordinary demographic transformation.

## 2. Definition and Staging of Chronic Kidney Disease in Adults

In 2002 the Kidney Disease Outcomes Quality Initiative (KDOQI) guidelines defined the criteria for the diagnosis of CKD [9]. The further classifications substituted the previous definitions of nephropathy, relying on a series of poorly definable descriptive parameters and gave greater importance to the early stages of kidney disease in order to identify the disease preciously. According to the KDOQI guidelines and subsequent Kidney Disease Improving Global Outcomes (KDIGO) modifications, CKD is characterized by a structural or functional dysfunction for ≥3 months. Glomerular filtration rate (GFR) and albuminuria are the two criteria utilized to classify CKD into “stages”. GFR divides kidney disease into five progressive stages while albuminuria identifies three additional categories for each level of kidney function. The combination of GFR (CKD stages, I–V) and albuminuria (A1–3) thresholds assumes also a prognostic significance, as classification in CKD staging predicts kidney survival [10]. However, three issues essentially limit the advantage of CKD classification in predicting the evolution of nephropathy and, above all, in planning an effective preventive strategy: assessment of albuminuria, methods for calculating GFR and the lack of a unanimously approved age-stratified CKD staging.

According to the 2009 KDIGO Controversies Conference report [11], albuminuria is a key criterion for the diagnosis of CKD. The magnitude of albuminuria may be easily misinterpreted because fluctuations are common in the real-word, and hypertension, cigarette smoking, inflammation and obesity may affect its excretion [12]. Furthermore, albuminuria may be overestimated in the elderly as reduced creatinine excretion secondary to the age-related decrease of muscle mass causes an increase in the urinary albumin/creatinine ratio. Lastly, to avoid overdiagnosis of CKD, the criterion of albuminuria should be met over at least 3 months of observation.

CKD prevalence estimations are influenced by population characteristics and different laboratory methods [13,14]. Ideally, to have an accurate estimate of GFR, it should be measured with nuclear medicine procedures, because formulae based on serum creatinine are characterized by several and well-known limitations [15]. Cystatin C provides an accurate alternative for measuring GFR. It is a reliable endogenous marker for the evaluation of kidney function compared to creatinine. Cystatin is not dependent on muscle conditions, therefore is more suitable in elderly patients with sarcopenia. Despite these advantages, its concentration is influenced by factors such as smoking, obesity, and inflammation [16,17]. The clearance of substances such as inulin, iohexol and iothalamate, allows a very precise measurement of renal function, excluding interfering variables such as age, body weight, muscle mass or inflammatory status from the calculation. Unfortunately, the availability of instrumentation in peripheral laboratory settings and lack of standardization potentially hamper comparisons across studies.

The Chronic Kidney Disease Epidemiology Collaboration (CKD-EPI) equation currently represents the most correct calculation method for estimating GFR in the general population. This equation overcomes the limit of the Cockcroft–Gault equation [18] (overestimation of GFR in obese people) [19] and MDRD (underestimation of GFR in people with normal or slightly reduced renal function [GFR between 60 to 100 mL/min]) [20].

The main limitation of the CKD-EPI equation is the tendency to overestimate GFR in the elderly. To overcome this discontinuity, which may have severe repercussions on renal function assessment and drug dose adaptation, a study conducted on white German participants aged >70 years with a mean measured GFR of 16–117 mL/min proposed the BIS-1 and BIS-2 GFR estimating equations [21]. These promising equations, albeit furnishing a precise and accurate tool to assess renal function in the elderly, still lack external validation studies against the KDIGO-recommended CKD-EPI equation. This latter also confirmed its superior performance when compared to the recent Lund–Malmö [22], FAS (Full Age Spectrum) [23] and CAPA (Caucasian and Asian Pediatric and Adult Subjects) [24] equations in the adult population.

In the absence of urinary alterations, the diagnosis of CKD is made by a GFR less than 60 mL/min. The use of a fixed threshold value across all age categories is undoubtedly a limiting element for the definition of CKD in the most extreme age groups of the population (i.e., young and elderly people). In these two groups, a similar value of GFR underlies a different prognostic value of kidney function, since projected life expectancies are poorly comparable. Based on these data, the classification of kideny function, embedded in a “rigid” staging system, may lead to inaccurate estimates of kidney outcomes. A classic example is the diagnosis of CKD in “healthy” old patients with a physiological decrease in kidney function.

**Figure 1:**
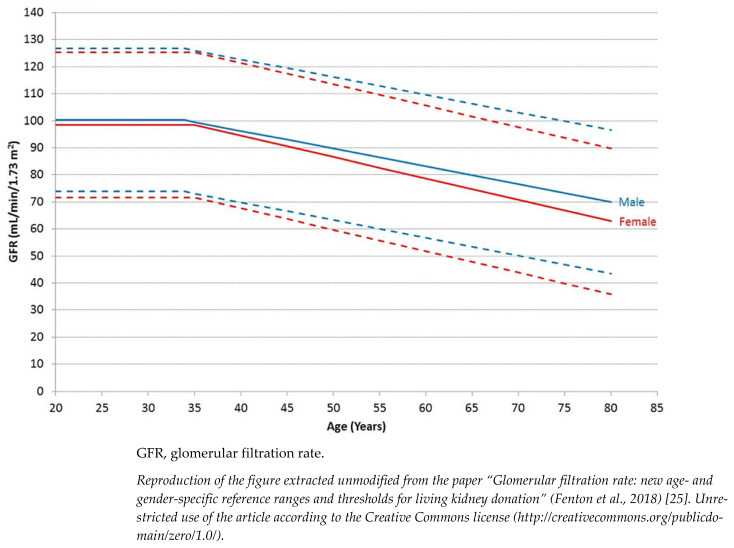
Age- and gender-specific GFR reference ranges. Data include pre-donation mean GFR from 2974 prospective living kidney donors from 18 UK renal centers performed between 2003 and 2015. Solid lines represent mean GFR and interrupted lines are two standard deviations above and below the mean.

The current classification of CKD indeed does not separate kidney disease from kidney senescence, a physiological phenomenon occurring after 40 years of age (Figure 1) [25]. In support of this theory, histological evaluation of kidneys from elderly donors confirms a non-specific and generalized involution of the renal parenchyma. Evaluation of kidney biopsy revealed nephroangiosclerosis, global ischemia, tubular atrophy and interstitial fibrosis as well as a considerable reduction in the total number of nephrons in the absence of a real compensatory adaptation [26]. The decline of the filtrate usually becomes significant after 40 years of age regardless of the ethnicity of the population examined. Beyond this age, the decline in GFR is constant and could reach the lower normal limit of 45 mL/min in subjects aged more than 65 years.

A meta-analysis conducted by the “CKD Prognosis Consortium” showed that the risk of ESKD and mortality is generally increased when GFR is substantially lower than 60 mL/min, but surprisingly, this threshold is lower in elderly patients [27]. Indeed, the elderly population with a GFR between 45 and 59 mL/min/1.73 m^2^, in the absence of urinary anomalies, tends rarely to progress towards ESKD (<1% at 5 years) [28].

**Figure 2:**
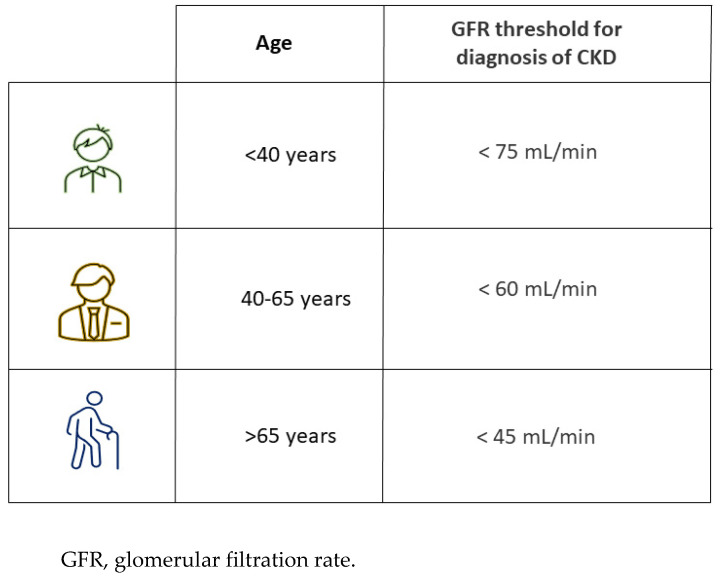
Age-related threshold for diagnosis of CKD.

Epidemiological studies have also reported that patients aged more than 65 years have a considerably higher risk of CKD progression only when the GFR is less than 45 mL/min. In support of the thesis, the “Renal Risk in Derby” study, conducted on 1741 people with a mean age of 72.9 ± 9 years and with an average GFR of 54 ± 12 mL/min/1.73 m^2^, confirmed that patients with stage IIIa of CKD have a mortality risk lower than CKD stage IIIb and IV and more importantly, these patients had a similar survival rate than the general population [29]. Based on these data, Delanaye et al. [30] proposed a CKD staging stratified according to three age categories: <40, 40–65 and >65 years. A GFR threshold of 75 mL/min should be considered “normal” for patients aged less than 40 years, 60 mL/min for individuals aged 40–65 years and 45 mL/min for the oldest (Figure 2).

In other words, nephrological evaluation of the elderly patient with a reduction of GFR can no longer depend on a laboratory reporting system that defines GFR > 60 mL/min as normal. The use of a fixed threshold at 60 mL/min may induce misinterpretation of renal function. For instance, GFR slightly greater than 60 mL/min is a strong negative predictor of renal and patient survival in young patients. On the contrary, GFR slightly below 60 mL/min without urinary alterations in a patient aged >65 years represents a physiological condition not subject to further diagnostic investigations.

The evaluation of kidney function is also key in the setting of living kidney transplantation as post-donation GFR should remain within normal range without affecting the donor’s survival and the recipient should receive a healthy graft not affected by CKD. The correct interpretation of the donor’s kidney function is complex and must take into account the physiologic reduction of GFR with aging as well as potential comorbidities and lifetime risk of developing ESKD after donation [31]. In parallel to the age-adapted threshold for diagnosis of CKD in the general population, UK guidelines for kidney transplantation have released advisory threshold GFR levels for living kidney donation. As expected, the GFR threshold for performing a safe living kidney donation decreases with aging. In donors aged >30 years, it can range from 80 to 58 mL/min in males and from 80–49 mL/min in females [32].

## 3. Epidemiology of CKD

According to current estimates, about 700 million people are affected by CKD worldwide. The worldwide prevalence of CKD stage I–V is estimated between 3% and 18%, with a higher prevalence in women than males in patients older than 40 years [33]. Recent estimates (2015-2018) of CKD in the United States (US) showed that the overall prevalence of CKD, defined as eGFR <60 mL/min/1.73 m^2^ or urinary ACR ≥30 mg/g, in the adult US population is 14.4%. Most of the CKD population (93.7%) is affected by stages I and II, namely, early stages of kidney disease characterized by a mild decrease in their GFR (>60 mL/min). The distribution of patients based on KDIGO risk categories indicates that 1.3% of the CKD population is at high risk to progress toward kidney failure and receive RRT. As aforementioned, CKD is common in elderly patients [4]. About 40% of subjects living with GFR < 60 mL/min are aged 65 years old or older [4].

Demographic characteristics, quality of healthcare, socio-cultural level of the population and methods used for the evaluation of renal function are the main factors influencing the rate of CKD [34]. The differences in the rate of CKD increase especially between populations with different socio-cultural differences. Age-adjusted CKD prevalence ranges between 5.5% in people living in Spain and 13.7% in those living in Russia [14]. One glaring and surprising example is the considerable difference in the rate of CKD that has been found between counties with similar socio-economic and cultural profiles such as Norway (3.3%) and northeast Germany (17.1%) [35]. However, these results need to be interpreted with caution because different factors contribute to these epidemiological disparities. First, most studies are conducted in single regions or cities and, therefore, are poorly representative of the entire national territory. Second, modifiable factors such as genetic susceptibility [36,37,38] and environmental background (i.e., dietary pattern, infections, air pollution) [39,40,41,42] may drive many risk factors for the development of CKD.

## 4. Risk Factors

Risk factors most closely related to the development of CKD in the general population are age (OR 1.06, CI 1.05–1.07), hypertension (OR 1.55, 1.23–194), cardiovascular disease (OR 1.9, CI1 0.47–2.42), smoking (OR 1.34, CI 1.05–1.72), diabetes (OR 1.98, CI1.59–2.46) and obesity (OR 1.42, CI 1.17–1.73) [43]. Other factors such as African–American ethnicity [44], male sex [45], familiarity [46], low birth weight [47], heavy metal exposure [48,49] and smoking [50] have been identified as further factors involved in the development of kidney disease. Diabetes and hypertension are the leading cause of CKD in the US, Europe and Asia [5], whereas HIV infection [51] and exposure to toxins or heavy metals [52] have a role in low-income countries.

Risk factors for CKD rarely act alone in the older people because aging increases the prevalence of multiple morbidities having synergistic effects on the development of CKD. Age is the main risk factor for CKD. About 11% of individuals aged more than 65 years without main comorbidities have CKD stage 3 or higher [53]. Besides age, hypertension and type 2 diabetes mellitus are the most significant risk factors for CKD. The synergetic effect of these two diseases is almost inevitable given their high prevalence (>50%) in adults aged ≥65 years [54,55]. Aging is also associated with other potentially adverse lifestyle factors, such as lower levels of physical activity, obesity and poor dietary quality which may exacerbate conditions such as insulin resistance and hypertension.

A large community-based retrospective cohort study of Japanese adults confirmed that older age was related to faster loss of kidney function. The main risk factors for worsening of kidney function in subjects aged ≥80 years were higher systolic blood pressure, proteinuria and current smoking [56].

A study based on the registry of “Prevention of Progressive Renal Insufficiency Project of Emilia-Romagna region (Italy)” conducted in a large cohort of patients with a mean age of (71.2 ± 12.9 years) and a mean GFR of 28.8 mL/min, regularly followed by nephrologists, documented that proteinuria, young age, diabetes and hyperphosphatemia are the leading risk factors for progression of CKD toward ESKD. It is interesting to note that late-stage CKD and advanced age are not strictly associated with a rapid progression of chronic kidney disease. Old patients, especially women, tend to have a slower progression compared to the young ones, who have a severe prognosis of the underlying kidney disease [57].

**Figure 3:**
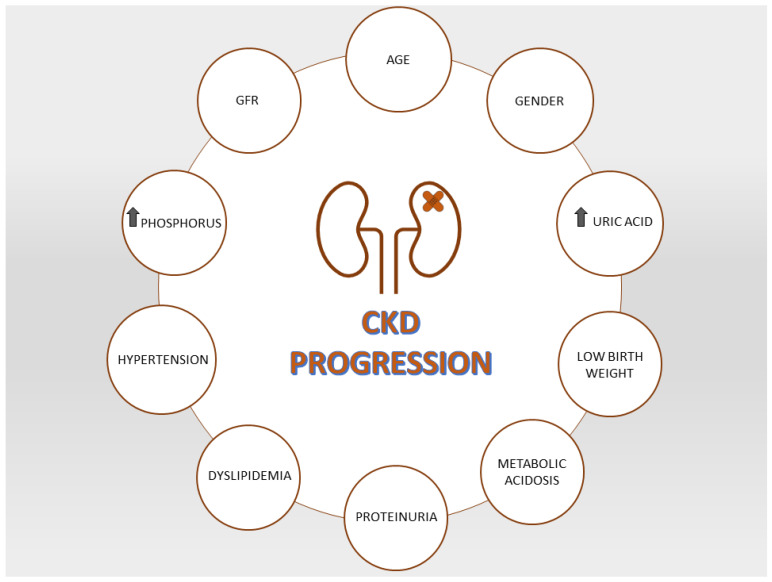
Risk factors for CKD progression.

The KDIGO guidelines classify the probability of progression of CKD into 4 risk categories: low, moderately high, high and very high [10]. In the real world, the progression of kidney disease relies on multiparametric variables taking into account age, comorbidities, etiology and rate of progression (Figure 3). The cumulative effects of all these factors classify patients as slow (≤5 mL/min) or fast progressors (>5 mL/min) [58]. All stages of kidney disease require assessment and control of risk factors. Adequate primary prevention strategies aimed to prevent harmful behaviors often occurring already in childhood or adolescence including smoking, incorrect diet and physical inactivity. The correction of pathological conditions strictly linked with the development of CKD (hyperglycemia, hypertension, dyslipidemia, obesity) requires a multispecialty approach (nephrologists, diabetologists and dieticians) whose goal is to avoid the rapid progression toward renal failure. Besides the routine care of CKD, the nephrologist has therefore the leading role to seek the support of other specialists for patients having risk factors for kidney progression.

## 5. Morbidity and Mortality of the CKD

Reduction of the nephron mass and therefore of the glomerular filtrate determines a rearrangement of the main homeostatic mechanisms regulated by the kidney. The first metabolic complications (hyperparathyroidism and anemia) develop for GFR values usually lower than 50 mL/min and other complications (acidosis, hyperphosphatemia and hyperkalemia) are observed for progressively lower values of the glomerular filtrate [59]. Nonmetabolic complications (neurological, dermatological and gastrointestinal pathology) are tardive pathological processes developing during the progression of the disease [60,61].

Cardiovascular disease is the most severe and frequent complication in patients with CKD. Mortality from cardiovascular disease is high for all stages of CKD, even though it is dramatically high in dialysis patients (15–30 times higher than the general population) [62,63,64]. Overall, the US Renal Data System Renal Data System 2018 Annual Data showed that patients with kidney disease have a double mortality index compared to their non-CKD counterparts.

In a population of older outpatients, CKD contributes significantly to multimorbidity patterns and it was rarely observed without any co-occurring disease. The most significant co-occurring pairs involving CKD included hypertension, anemia, cardiovascular disease, hip fracture and, to a lesser extent, hearing impairment, diabetes and cancer [65]. Generally, a subject with kidney disease, especially older than 65 years, has high rates of hospitalizations mainly related to the complications of renal failure and the various comorbidities associated with this disease. The probability of hospitalization of a patient with kidney disease is 147% higher than the unaffected counterpart [66]. The main causes of hospital admission are cardiovascular events (19.7%) and infections (17.8%) [67]. As expected, elderly patients and advanced stages of kidney disease have a higher mortality rate than that of the age-matched general population. The US national registry of patients with ESKD revealed that median survival after dialysis initiation was 24.9 months for patients 65 to 79 years of age; 15.6 months for patients 80 to 84 years of age; 11.6 months for patients 85 to 89 years of age; and 8.4 months for patients 90 years of age or older [68]. Besides age, nonambulatory status hypoalbuminemia, congestive heart failure and being underweight were associated with very high mortality rates at dialysis initiation [68].

## 6. The Cost of CKD

CKD poses substantial expenses to the healthcare system [69]. In the US, it is estimated to be about 33.8% of the annual budget for the Medicare population (subjects aged >65 years or with severe comorbidities). The growth of healthcare costs for a subject with CKD has primarily been driven by a rise in the number of new cases, particularly those in the earlier stages (CKD stages 1–3) [70].

An economic cost analysis in Alberta, Canada, reported that expenditure for CKD was higher for patients with lower GFR, more comorbidity, lower education and socioeconomic position. Hospitalization, drugs, physician and ambulatory care accounted for 38%, 35%, 14% and 13% of the annual total costs, respectively [69].

Total costs (direct and indirect) are highly variable among countries. For the Italian healthcare system, it is $530 per year in stages I and II, $1050 in stage III, $4010 in stage IV and $10,612 in stage V. The costs of renal replacement therapy (RRT) are $62,500 per patient-year for hemodialysis and about $45,900 per patient-year for peritoneal dialysis. Kidney transplantation was estimated to cost $61,520 per patient-year the first year and $17,700 from the second year [71]. A more detailed analysis shows that direct non-medical and indirect costs are about 50% of the expenditure in stages IV and V, whereas healthcare costs are 50% of direct medical costs [72]. The cost of care is slightly different in the US. It is interesting to note that the expenditure for transplant patients per year ($35,817) is far lower than spending for hemodialysis ($91,795) and PD ($78,159) [70]. Annual per-person costs for subjects aged 66 years and older with CKD (not including costs for ESKD) accounted for $21,508 and were approximately twice those for persons without CKD [4]. 

The economic management of CKD also includes direct medical costs (laboratory tests, specialist visits, hospitalizations, drugs, disposable medical devices), direct non-medical costs (transport, specialist dietary visits, low protein food and domestic help) and indirect costs (loss of productivity of the affected patient and/or his caregivers). An additional expense, usually not considered in the management of CKD, is the employment of an external caregiver to help the frails subjects at home and to perform home dialysis in non-self-sufficient patients with ESKD. CKD gradually manifests with a decline in cognitive functions, sleep disturbances, pain, as well as suboptimal control of underlying comorbidities. Furthermore, the chronic and debilitating nature of the disease can invalidate frail patients, resulting in extreme cases of loss of patient autonomy.

## 7. ESKD Trend

The incidence of ESRD has reached the plateau both in the US [4] and Europe [73]. In Asia data are limited, but recent epidemiological studies reported a concerning increase in CKD prevalence in India [74] and Nanjing, a high-populated city in China [75]. Conversely, countries with valid welfare regimes such as Finland or Switzerland showed a decreasing trend in ESKD incidence compared to the past [73].

In Europe, incidence of patients on hemodialysis, peritoneal and pre-emptive transplantation is 85%, 11% and 4%, respectively. The rate of kidney transplantation is well-represented among prevalent patients, as 37% of ESKD received kidney transplantation [76].

In the US there is a higher incidence of ESKD than in Europe. The main modalities of renal replacement therapy for incident patients are hemodialysis (85.1%), peritoneal dialysis (11.5%) and pre-emptive renal transplantation (3%). Once dialysis started, kidney transplantation became a valid option as the rate of this RRT modality rise to approximately 30% within 1 year in patients being placed on the waitlist. Also, home hemodialysis, endorsed by national policy, has a greater prevalence than in Europe (1.9% vs. 0.2%) [3,4].

Similar to CKD stages I–V, ESKD requiring dialysis prevalence has a different global distribution. A key issue is the “access” to RRT. Different regulatory policies and social inequities may explain disparities in the rate of ESKD. In Europe, the countries with the highest prevalence of ESKD (i.e., Spain, Belgium, Italy) have “liberal” access to dialysis care compared to other countries with comparable welfare [73]. In these countries, age and probably the bulk of comorbidities may be a not limiting factor for commencing dialysis compared to other places. Indeed, the advanced median age (over 70 years) of patients who start dialysis in some regions of these countries, reflects a more “unconditional” access to RRT. Analysis of the ERA-EDTA registry document that over 50% of the incident population on RRT is 65 years of age or older.

## 8. Kidney Transplantation in the Elderly Population

Kidney transplantation is considered the preferred treatment option for ESKD as it offers a survival advantage over dialysis for the majority of patients including older patients [77,78]. Over the last two decades, an increasing number of elderly patients have received a single or dual cadaveric kidney transplantation from expanded criteria donors, namely, donors aged > 65 years most often affected by comorbidities [79]. Analysis of the renal registry in Catalonia, an autonomous region of Spain with one of the highest transplantation rates worldwide [80], shows that kidney transplant activity mainly involves older patients, notably half of the new patients on RRT are older than 70 years and more than half of donors are over 60 years old [81]. However, assessing the eligibility for kidney transplantation for older patients often involves tackling complex issues including frailty, cognitive impairment and comorbidities. For these reasons, older candidates for kidney transplantation should be screened more carefully than younger recipients for cardiovascular disease and cancer. After kidney transplantation, older transplant recipients tend to experience more infectious complications and a higher risk of transplant loss from rejection compared to younger patients. Furthermore, these patients should be informed of the increased risk of death during the first weeks after transplantation compared to waitlisted patients who remained on dialysis [82].

The critical shortage of organ supply has also stretched the limits of organ donation in terms of age and comorbidities. The use of extremely elderly donors raises questions about the real potential benefit obtained from these organs. A study conducted in Spain showed that recipients receiving a kidney from donors aged >80 years had lower mortality rates than those remaining on dialysis. As expected, graft survival was significantly lower than deceased donor kidneys aged between 60 and 79 years [82].

In parallel, recipients of living donor kidneys aged ≥70 years had a significantly higher rate of graft loss compared with recipients of younger living donor kidneys aged 50 to 59 [83]. Nevertheless, kidney transplantation from a healthy older live donor remains a reasonable option compared to the well-documented risks of remaining on a waitlist. Avoiding the waitlist and proceeding directly to pre-emptive kidney transplantation from living donors lead to a series of advantages for the recipients and society, including better patient survival and cost reduction of long-term hemodialysis [84].

## 9. Perspectives

Disease registries offer the advantage of understanding the trend of CKD in a given setting, verifying the effect of the implemented actions and organizing plans for CKD prevention. A useful tool to assess the quality of nephrology care and verify the effectiveness of preventive measures against kidney disease is to monitor the trend of diabetic nephropathy. This estimate, in part biased by different genetic and dietetic backgrounds of the populations, reflects the availability and efficiency of outpatient care services in terms of prevention, education, socioeconomic status as well as the interconnection between different providers such as nephrologists, diabetologists and primary care physicians.

Given the high prevalence of CKD in the elderly, KDIGO in 2006 suggested that CKD screening should be offered to patients with risk factors and those over the age of 60 years [85]. Taking the perspective of the nephrologist, older patients with a new diagnosis of CKD should be referred to the nephrology service with a series of diagnostic examinations including the value of serum creatinine, urinalysis, proteinuria, albuminuria and, if possible, urinary system ultrasound. Causes of CKD need to be carefully investigated as kidney injury often is related, especially in the elderly, to extra-renal disorders (hematological disorder, cancer, diabetes, cardiovascular disease) [86]. Kidney biopsy should be judiciously performed in elderly patients with acute kidney injury (AKI) and CKD regardless of age. Histopathological evaluation is not only useful to establish a diagnosis but can be also informative on the irreversibility of lesions, overall prognosis [87] and increased risk of extrarenal lesions. However, kidney biopsy is rarely performed in elderly patients because it is thought that immunosuppressive treatment may outweigh the clinical benefit in this population [88]. Data from the literature revealed that one-third of elderly patients with AKI had a diagnosis of pauci-immune glomerulonephritis [87] and that patients aged >80 years with biopsy-proven ANCA–associated vasculitis had a better outcome than their non-treated counterparts [89]. Furthermore, kidney biopsy is not associated with major complications in this group of patients [90] except for an increased incidence of gross hematuria documented in a small cohort of patients aged >60 years [91].

Care of CKD stage IIIa without proteinuria and tendency toward progression can be effectively perpetuated by the primary care physician. The best therapeutic management of these patients is based primarily on the reduction of metabolic and cardiovascular risk factors including blood pressure control, lifestyle and balanced diet as depicted in Table 1. In particular, renin-angiotensin-aldosterone system (RAAS) inhibitors such as angiotensin-converting enzyme and angiotensin II receptor blockers have shown undeniable renoprotective effects, especially in proteinuric and diabetic nephropathy [92,93]. Their use for older patients with CKD remains unclear because they can cause life-threatening hyperkalemia and AKI [94]. Blockade of the aldosterone pathway is another attractive therapeutic intervention to retard the progression of CKD induced by aldosterone-mediated fibrosis [95,96]. Finerenone, a novel selective mineralocorticoid receptor antagonist, is an antifibrotic agent that showed promising results in lowering the risks of CKD progression and cardiovascular events in patients with CKD and type 2 diabetes [97]. As vascular calcification in patients with kidney disease is no longer seen as a passive process resulting from an elevated calcium-phosphate product [98], vitamin D receptor agonist therapy seems to play a decisive role in contrasting vascular calcification, a dire complication of CKD closely associated with cardiovascular mortality [99]. A new class of antidiabetic agents, sodium-glucose cotransporter 2 (SGLT2) inhibitors have shown favorable effects on kidney outcomes in patients with type 2 diabetes [100,101]. Recent evidence revealed that SGLT-2 inhibitors act independently of their blood glucose–lowering effect on diabetes. Indeed, dapagliflozin significantly lowered albuminuria [102] and reduced the risk of progression of CKD and death from renal or cardiovascular causes, regardless of the presence or absence of type 2 diabetes [103]. Lastly, prevention of drug-induced nephrotoxicity (aminoglycosides, antiviral, non-steroidal anti-inflammatory drugs, iodinated contrast medium) is crucial in elderly patients who carry a high risk of AKI [104].

A nephrology intervention should also focus on patients with a high risk of progression. A dedicated outpatient service for patients with advanced CKD (>20 mL/min) is advisable to monitor closely kidney function and establish an educational program on RRT including pre-emptive kidney transplantation [126]. According to national policies, ESKD patients should start examinations to confirm eligibility for pre-emptive kidney transplantation, as this modality is the first option among RRTs. Dialysis should be tailored to the needs and comorbidities of the subject. Implementation of home dialysis, albeit hampered by multiple barriers, has the great advantage to increase patients’ well-being by reducing hemodialysis-related hospitalization. A therapeutic strategy based on preservation of health-related quality of life should be offered to subjects with a short life expectancy (within six months or less) through a conservative (nondialytic) management of renal failure [127]. Elderly patients on dialysis are more likely to be hospitalized, are at risk for infectious and cardiac complications, receive ICU care and die in the hospital in the last 30 days of their life [128,129]. There is also growing evidence that RRT provides little benefit to frail and comorbid patients with progressive CKD. This is especially true for patients with ischemic heart disease, congestive heart failure or advanced atherosclerosis. Findings from the 2015 Canadian Organ Replacement Register showed that patients aged ≥75 years on maintenance RRT had a 5-year survival of only 27% [130]. A single-center retrospective study conducted in Nieuwegein, Netherlands, found that starting dialysis in people over 80 years of age did not furnish a significant advantage compared to patients treated conservatively [131].

Individualization of the appropriate treatment is, therefore, crucial to improving patient’s quality of life. Nevertheless, there is a limited understanding of existing prognostic tools because they lack external validation [130]. The absence of prognostication model makes discussion on the prognosis of the patient extremely stressful for the nephrologist, especially when this information is addressed to pre-dialysis patients and patients on dialysis who lack decision-making capacity. Nephrology providers felt also unprepared to discuss conservative management of ESKD or end-of-life care with patients aware of their poor prognosis but amenable to receiving invasive dialytic treatment to alleviate the uremic symptoms. It is worth highlighting that advanced care planning, including conservative management of ESKD or withdrawal from dialysis, should be managed by a multidisciplinary team integrated into CKD clinics. The dedicated team should provide care for these patients, help to coordinate a smooth transition of care in another structure (e.g., hospice, home care) and coordinate therapeutic strategies to mitigate the uremic symptoms (e.g., pain, dyspnea, pruritis, agitation, secretions, nausea and vomiting) manifesting in the late stage of ESKD.

## 10. Conclusions

In the setting of the current demographic change, the prevalence of CKD and advanced stage of CKD will inevitably tend to increase over time, with significant consequences on the national healthcare systems. Prevention and appropriate management of risk factors for CKD in the young and middle-aged population is key to limiting a further increase in CKD prevalence among the elderly. Kidney dysfunction is common in the elderly population as renal senescence is associated with a progressive and relentless GFR decrease. The diagnosis of CKD based on a fixed threshold is necessarily associated with overdiagnosing of CKD in older people. A context of age-adapted definition is therefore useful to establish effective reduction of kidney function. Once the diagnosis of CKD has been confirmed, coordination with other specialists and with primary care physicians is fundamental to delivering the appropriate care. Primary care physicians may provide the bulk of care for patients with “indolent” CKD and furnish the nephrologist with the key elements to understand the etiology and risk factors for the progression of CKD. A high level of awareness of kidney disease should be pursued among healthcare workers since late diagnosis and consequently “late referral”, is closely associated with lower survival, worse quality of life and high use of economic resources. 

## Figures and Tables

**Table 1:** Therapeutic interventions for limiting the progression of chronic kidney disease.

Therapeutic Intervention	KDIGO Recommendation	Results of Randomized Controlled Trial/Scientific Evidence
Slowing down the progression of CKD	No specific recommendations on the inhibition of the renin-angiotensin-aldosterone system	Inhibition of renin-angiotensin-aldosterone system [105,106] SGLT-2 inhibitor [103] Finerenone [69]
Diabetic patient with CKD	Inhibition of the renin-angiotensin-aldosterone system	Inhibition of renin-angiotensin-aldosterone system [66] SGLT-2 [100,101]
High blood pressure without microalbuminuria	Goal: ≤140/90 mmHg	<130/80 mmHg [107,108]
Diabetes	Glycated hemoglobin ≤7.0% (53 mmol/mol)	No benefit of intensive control of glycated hemoglobin [109,110]
Dietary intake of protein	0.8 g/kg/die in patients with diabetes or GFR <30 mL/min/1.73 m^2^	Low protein diet leads to better metabolic control [111]
Dyslipidemia	Statin or statin/ezetimibe: -in adults aged > 50 years with CKD regardless of cholesterol levels -in adults aged < 50 years only if they have had CV events, have diabetes or have a CV risk of more than 10% at 10 years. Statin is not recommended in dialysis patients; if already in therapy at the time of dialysis start, continue the current therapy	Lower LDL by >50% in those <75 years and a high or moderate intensity statin in those over age 75 [112] LDL-cholesterol <70 mg/dL in patients at high CV risk and <55 mg/dL in patients at very high CV risk [113]; no benefit in dialysis patients [114]
Acid-base balance	Serum bicarbonate ≥22 mEq/L	Bicarbonate supplementation (serum value ≥23 mEq/L) slows the rate of progression of CKD and improves nutritional status [115]
Hyperuricemia	No recommendations	Reduce uric acid below the saturation point (<6 mg/dL) [116] Possible implication of hyperuricemia on progression of CKD [117,118].
Physical activity	Allowed (30 min 5 times a week)	Slight improvement in GFR [119,120]
BMI	Goal: BMI 20 to 25	Weight loss can reduce glomerular hyperfiltration proteinuria and albuminuria [121,122]
Dietary salt intake	Na <90 mmol/daily (<2 g/daily) NaCl < 5 g/daily	Sodium restriction (goal 60–80 mmol/day)reduce cardiovascular risk for CKD progression [123] and proteinuria [124]
Smoking	Avoiding	Smoking is associated with CKD progression [125]

## Data Availability

Not applicable.
